# Design and Evaluation of an Edge AI-Enabled Low-Power Magnetic Sensor for Real-Time Road Traffic Monitoring

**DOI:** 10.3390/s26165315

**Published:** 2026-08-21

**Authors:** Michal Hodoň, Peter Šarafín, Lukáš Formanek, Andrea Kociánová

**Affiliations:** 1 Department of Technical Cybernetics, Faculty of Management Science and Informatics, University of Žilina, Univerzitná 8215/1, 010 26 Žilina, Slovakia; peter.sarafin@fri.uniza.sk (P.Š.); lukas.formanek@fri.uniza.sk (L.F.); 2 Department of Highway and Environmental Engineering, Faculty of Civil Engineering, University of Žilina, Univerzitná 8215/1, 010 26 Žilina, Slovakia; andrea.kocianova@uniza.sk

**Keywords:** magnetic sensor, Edge AI, TinyML, road traffic monitoring, vehicle detection, vehicle classification, RM3100, low-power embedded system, intelligent transportation systems

## Abstract

Road traffic surveys require sensing systems that can be deployed rapidly without modifying the road surface or requiring a permanent power connection. This paper presents the design, embedded implementation, and evaluation of a low-power roadside magnetic sensor that performs vehicle-event detection and classification directly at the edge. The sensing node integrates two RM3100 three-axis magnetometers (PNI Sensor, Santa Rosa, CA, USA) with an NXP MK22FN512VLH12 microcontroller (NXP Semiconductors N.V., Eindhoven, The Netherlands) based on a 120 MHz Arm Cortex-M4F core with 512 kB Flash and 128 kB SRAM. Magnetic-field data are acquired at 250 Hz and processed locally using baseline removal, low-pass filtering, signal-energy calculation, and peak-based event detection. Detected magnetic signatures are classified using an integer-quantised one-dimensional convolutional neural network implemented directly on the microcontroller. The model processes four synchronised 512-sample channels representing the three magnetic-field axes and their combined signal energy. Model development was supported by approximately 50,000 annotated events obtained from 36 h of real-world traffic measurements at eight locations. The selected model achieved an overall classification accuracy of 91.1% for the considered operational categories. The implemented network requires 288,128 multiply–accumulate operations per inference, while its quantised weights and biases occupy approximately 23 kB of Flash memory. Complete three-axis event signatures are stored locally for subsequent verification, whereas only the timestamp and predicted vehicle category are transmitted through the wireless interface. Based on the capacity of the applied LiFePO_4_ battery and the estimated consumption of the implemented hardware, the expected autonomous operating period is approximately 41 days. The results demonstrate the feasibility of integrating magnetic sensing, embedded signal processing, and Edge AI on a conventional resource-constrained Cortex-M4 platform for non-invasive road traffic monitoring.

## 1. Introduction

Accurate road traffic data are essential for transport infrastructure planning, traffic management, road safety assessment, emission modelling, and the development of intelligent transportation systems. Traffic surveys provide information about traffic volume, vehicle composition, direction of travel, speed, and temporal distribution. Such information is required not only for the design and reconstruction of road infrastructure but also for the evaluation of traffic-control measures and the preparation of noise and environmental impact studies. Depending on the application, traffic monitoring may be performed continuously at permanent measurement stations or temporarily at selected locations.

Recent research demonstrates that road traffic can be monitored using a wide range of sensing technologies combined with intelligent data-processing methods. The study presented in [1] proposed an iterative self-training segmentation method for scalable traffic monitoring based on distributed acoustic sensing, in which vehicle-induced vibrations recorded along optical fibres were segmented and subsequently used for traffic analysis. The potential of distributed acoustic sensing for real-time traffic monitoring was also demonstrated in [2], showing that existing fibre-optic infrastructure can provide spatially distributed information about passing vehicles. To improve the extraction of vehicle-related features from such vibration signals, the authors of [3] applied a superlet-based transformation capable of enhancing transient vehicle responses while suppressing background noise. Another infrastructure-integrated approach was presented in [4], where a piezoresistive asphalt–concrete sensor was developed to detect traffic loads directly from the mechanical response of the pavement. In contrast, the modular architecture proposed in [5] employed 3D LiDAR sensors for non-invasive vehicle detection and spatial characterisation without relying on conventional optical cameras. A deep learning-based UAV framework combining vehicle detection, tracking, and traffic-state prediction from aerial imagery was introduced in [6]. From the perspective of edge processing, the semantic image-communication methods proposed in [7] locally extracted and transmitted only relevant information about the monitored traffic scene, thereby substantially reducing the amount of data transferred to a remote processing system. Collectively, these studies illustrate the transition from conventional traffic detectors towards intelligent, data-driven monitoring systems. Nevertheless, many of the proposed solutions still depend on sensors integrated into the transport infrastructure, high-volume optical or vibration data, or comparatively powerful processing platforms.

For temporary roadside surveys, the choice of sensing technology is governed not only by detection accuracy but also by installation effort, data volume, privacy, environmental sensitivity, energy demand, and maintenance requirements. Camera systems provide rich semantic information but require image acquisition and comparatively intensive processing and may raise privacy concerns; LiDAR and radar reduce some illumination-related limitations but generally increase sensing cost and processing complexity. Acoustic and distributed-acoustic approaches can cover extended road sections, although their performance depends on the acoustic environment or the availability and coupling of suitable fibre infrastructure. Magnetic sensing provides a complementary option because it measures local perturbations of the geomagnetic field, generates low-bandwidth time-series data, operates independently of scene illumination, and can be combined with low-power embedded processing. Its principal limitations are the rapid reduction of disturbance amplitude with increasing sensor-to-vehicle distance and its sensitivity to installation geometry, nearby ferromagnetic structures, and vehicle trajectory [1,2,3,4,5,6,7,8,9,10,11]. These trade-offs motivate the roadside magnetic architecture considered in this work; the proposed approach is intended as a complementary solution rather than a universal replacement for camera-, radar-, LiDAR-, acoustic-, or pavement-embedded systems.

The present work deliberately builds on the authors’ earlier studies [12,13,14,15], and the revised manuscript explicitly distinguishes reused methodology from the new contribution. The earlier work addressed sensor-selection requirements, comparative magnetometer evaluation, traffic-signal processing, multimodal synchronisation, annotation, and dataset construction. Those elements are not claimed here as new. The novelty of the present study is the system-level hardware/software co-design that integrates the previously developed sensing and detection methodology into a custom roadside node. The new integration combines event-driven embedded processing, an integer-quantised 1D CNN executed directly on an NXP MK22 microcontroller (NXP Semiconductors N.V., Eindhoven, The Netherlands), local retention of verification data, selective wireless transmission, and an embedded resource and energy analysis. In other words, the contribution is the transition from separately validated sensing and dataset components to a complete resource-constrained Edge-AI implementation operating at the measurement location.

Despite the progress achieved in magnetic vehicle sensing, an important engineering-scientific question remains insufficiently addressed for the developed platform. Specifically, can the complete chain from magnetic acquisition and preprocessing to event detection and multiclass inference be executed directly on a conventional low-power Cortex-M4 microcontroller without an external AI accelerator or continuous raw-data transmission? The present study therefore focuses on the computational structure, parameter memory, event-driven execution, classification performance, communication strategy, and battery-operability implications of this embedded integration, while treating the previously published sensor-selection and dataset-generation procedures as enabling prior work rather than new results.

The main contributions of this work can be summarised as follows:

- The design and custom implementation of a complete roadside sensing node, including the PCB-level integration of magnetic sensing, embedded processing, local storage, wireless communication, and autonomous power subsystems.
- The deployment of a lightweight, integer-quantised 1D CNN directly on the NXP MK22FN512VLH12 (Arm Cortex-M4F) without a dedicated AI accelerator or external inference computer.
- The reuse of a previously published and manually verified real-traffic dataset [15] specifically to evaluate the new embedded inference chain; dataset creation itself is not presented as a contribution of this manuscript.
- A common-subset comparison of conventional classifiers and a compact temporal CNN, followed by implementation of the selected model as custom C inference code with event-triggered execution.
- The characterisation of the embedded model in terms of parameter storage and multiply–accumulate count, together with a communication strategy that transmits only compact event results and an explicitly identified engineering estimate of battery-powered autonomy.

To address this research gap, the present study aims to design, implement, and evaluate a complete low-power magnetic traffic-monitoring system capable of performing vehicle-event detection and classification directly on a resource-constrained embedded platform. Particular attention is paid to the integration of the sensing, signal-processing, and classification components, the implementation of the quantised 1D CNN on the NXP MK22FN512VLH12 microcontroller, and the practical applicability of the resulting roadside prototype.

The remainder of the paper is organised as follows. Section 2 presents the overall architecture, hardware implementation, and magnetic sensing subsystem of the proposed traffic-monitoring platform. Section 3 describes the magnetic signal-processing and vehicle-detection procedure, dataset preparation, and evaluation of the investigated vehicle-classification approaches. Section 4 presents the embedded implementation of the selected neural network model on the MK22 microcontroller, including input quantisation, local data storage, and wireless transmission of the classification results. Section 5 compares the developed prototype with selected commercial traffic counters and discusses its practical advantages and limitations. Finally, Section 6 summarises the main conclusions and outlines directions for future research.

## 2. System Overview

The proposed system was designed as a compact and autonomous roadside device for temporary and long-term road traffic monitoring. Its primary purpose is to detect passing vehicles, classify them into predefined categories, and store the resulting traffic records without requiring continuous transmission of raw sensor data to an external processing platform. The sensor can be installed beside the monitored road without modifying the road surface, which simplifies its deployment and allows it to be relocated between different measurement sites.

The system consists of four principal subsystems: a magnetic sensing subsystem, an embedded processing unit, a data storage and communication subsystem, and an autonomous power supply. The magnetic sensing subsystem is based on the RM3100 three-axis magnetometer and continuously measures changes in the local magnetic field caused by passing vehicles. The embedded processing unit is built around the NXP MK22FN512VLH12 microcontroller and performs sensor control, data acquisition, signal preprocessing, vehicle-event detection, preparation of the classification input, and execution of the vehicle-classification model. The resulting traffic records are stored locally and can subsequently be transferred to a user device through the available communication interface. The complete system is powered by a rechargeable LiFePO_4_ battery.

Unlike conventional acquisition systems that continuously transmit raw magnetic measurements, the proposed architecture performs the principal data-processing operations directly on the embedded device. Raw magnetic samples are first filtered to suppress baseline drift and measurement noise. A vehicle-detection algorithm subsequently identifies signal intervals corresponding to potential vehicle passages. For each detected event, a fixed-length signal window is generated and evaluated by the embedded classification model. During standard Edge-AI operation, the event timestamp, predicted vehicle category, and complete three-axis event signature are retained locally on the SD card, whereas only the timestamp and predicted category are transmitted through the wireless interface. The locally stored signatures support subsequent verification, error analysis, and model development without requiring continuous wireless transmission of raw data.

**Figure 1:**
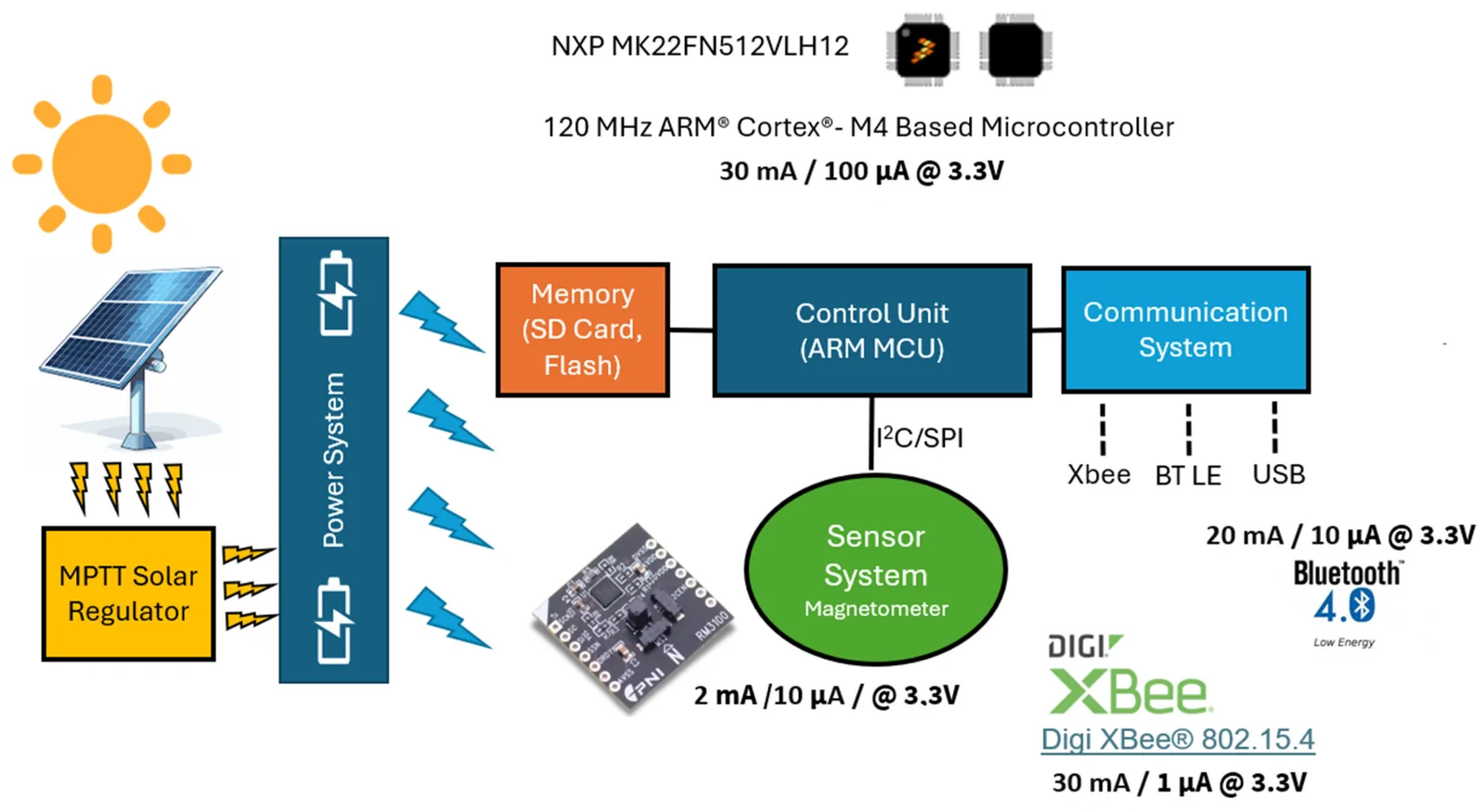
Hardware architecture of the proposed low-power magnetic sensor node.

The hardware architecture of the proposed sensor node is shown in Figure 1. The central processing element is the NXP MK22FN512VLH12 microcontroller, which communicates with the magnetic sensing subsystem through an I^2^C or SPI interface. The acquired and processed data are stored in local Flash memory or on an SD card. Communication with external devices is provided through USB, Bluetooth Low Energy, or a Digi XBee 802.15.4 wireless module. All system components are supplied by a common power subsystem integrating rechargeable energy storage, a photovoltaic panel, and an MPPT solar regulator. This architecture enables autonomous operation while allowing individual subsystems to be activated or placed into a low-power state according to the current operating mode.

As illustrated in Figure 1, the sensor node is organised around the NXP MK22FN512VLH12 microcontroller, which coordinates magnetic data acquisition, local data processing, memory access, and communication. The modular architecture was selected to support different operating scenarios, ranging from short-term traffic surveys using battery power to longer autonomous deployments assisted by photovoltaic energy harvesting.

The individual hardware components were selected with respect to the requirements of roadside traffic surveys, particularly low power consumption, sufficient computational performance, compact dimensions, modularity, data-storage capacity, and the possibility of autonomous operation. The NXP MK22FN512VLH12 microcontroller was selected as the central processing unit because its 120 MHz Arm Cortex-M4F core provides sufficient computational performance for magnetic data acquisition, digital signal processing, and local execution of the classification algorithm. At the same time, the microcontroller offers several low-power operating modes and integrates the communication interfaces required by the remaining system components. Its 512 kB Flash memory and 128 kB SRAM provide sufficient resources for the application firmware, signal buffers, and the embedded classification model without requiring an external high-performance processing platform. The RM3100 magnetometer was selected on the basis of the authors’ previous comparative studies [12,13,14]. In laboratory and real-traffic experiments, it provided the most favourable combination of sensitivity, low measurement noise, output data rate, and vehicle-detection capability among the evaluated digital-output magnetometers. Its three-axis output enables the complete magnetic signature of a passing vehicle to be recorded, while its configurable measurement parameters allow its performance and power consumption to be adapted to the requirements of the application. Communication through the I^2^C or SPI interface also enables straightforward integration with the selected microcontroller. Local Flash memory and an SD card were included to support different operating scenarios. Internal or external Flash memory can be used for system configuration, calibration parameters, the classification model, and short-term event storage. The SD card provides substantially higher capacity for long-term traffic records and, when required, raw magnetic signatures. This is particularly important during experimental measurements, algorithm validation, and the creation of new datasets. During standard Edge-AI operation, the SD card stores the event timestamp, predicted vehicle category, and corresponding three-axis magnetic signature. Wireless communication is limited to compact event messages containing the timestamp and predicted category, thereby reducing the amount of transmitted data. Multiple communication interfaces were included because the system is intended both for experimental development and practical roadside deployment. USB provides a reliable wired interface for firmware development, service operations, and high-speed data download. Bluetooth Low Energy enables local configuration and wireless transfer of measurement results using a nearby mobile device while maintaining comparatively low energy consumption. The Digi XBee 802.15.4 module provides a longer-range wireless connection and supports the integration of the sensor into a distributed wireless sensor network. The modular communication architecture allows currently unused interfaces to be disabled or placed in a low-power state. The power subsystem combines rechargeable energy storage with photovoltaic energy harvesting and an MPPT regulator. A battery enables continuous operation during periods without sufficient solar radiation, while the photovoltaic panel can replenish the stored energy and extend the deployment duration. The MPPT regulator was included to extract the available energy from the solar panel efficiently under changing illumination conditions. This combination is particularly suitable for roadside locations where a permanent electrical connection is unavailable and where frequent battery replacement would increase maintenance requirements and operating costs. Since the selected modules operate with substantially different active and low-power currents, their operating states are controlled independently. High-consumption components, particularly the wireless communication modules and SD card, are activated only when required, whereas magnetic sensing and low-complexity event detection remain active during traffic monitoring. This operating strategy reduces the average power consumption of the complete sensor node while preserving its sensing and communication functionality.

### 2.1. Hardware Architecture

**Figure 2:**
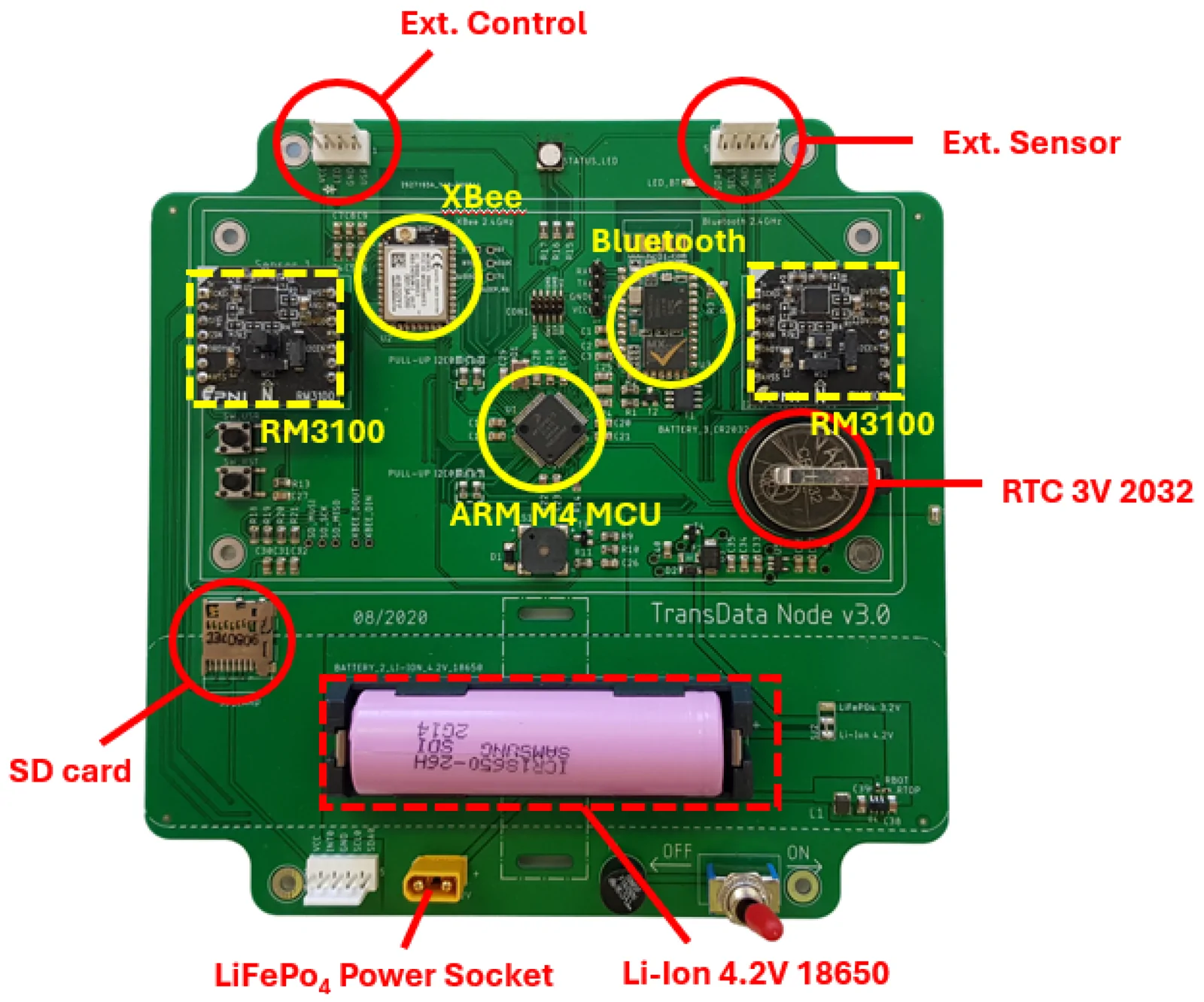
Hardware implementation of the low-power magnetic sensor node.

The functional architecture presented in Figure 1 was implemented as a custom printed circuit board integrating the principal sensing, processing, storage, communication, and power-supply components. The hardware implementation of the sensor node is shown in Figure 2. The central part of the board contains the NXP MK22FN512VLH12 microcontroller, which coordinates the operation of the remaining subsystems. Two RM3100 magnetometer modules are installed on opposite sides of the printed circuit board, while additional connectors allow an external sensor and an external control element to be connected when required.

Although the magnetometers, microcontroller, storage medium, and radio modules are commercially available components, the traffic-monitoring device is not an off-the-shelf assembly. The PCB integration, sensor interfaces, power-domain control, acquisition and buffering firmware, event-detection logic, per-channel quantisation, CNN inference routine, SD-card event-record structure, and compact wireless message format were developed specifically for the proposed platform. This customisation enables the complete processing chain to remain on a 512 kB Flash/128 kB SRAM microcontroller and avoids continuous transmission of raw magnetic data.

The communication subsystem integrates a Digi XBee 802.15.4 module and a Bluetooth Low Energy module directly on the main board. The XBee module enables wireless integration of the device into a distributed sensor network, whereas Bluetooth Low Energy provides local communication with a nearby configuration or data-download device. A removable SD card is used for storing traffic records and, during experimental measurements, raw magnetic data. The board also contains a real-time clock with an independent CR2032 backup battery, ensuring that timestamps are retained when the main power source is disconnected or discharged.

The power section supports different energy-source configurations. A dedicated power connector allows an external LiFePO_4_ battery subsystem to be connected [16], while the board also contains a holder for a single 18,650 lithium-ion cell. This modular arrangement was introduced to support both compact short-term measurements and longer deployments requiring a higher-capacity external battery. The selection of the final energy source depends on the required operating time and whether photovoltaic energy harvesting is available at the measurement location.

**Figure 3:**
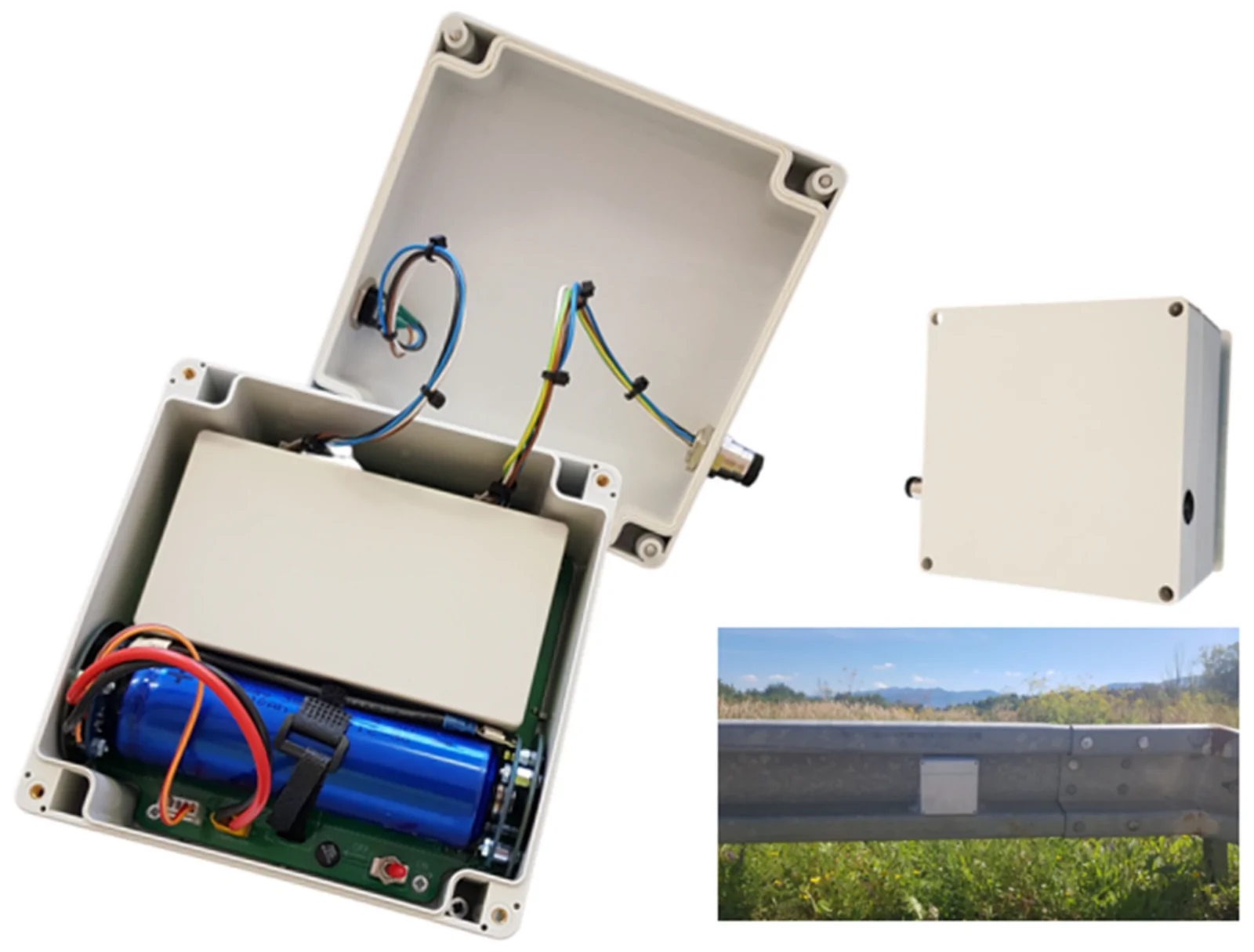
Mechanical implementation of the low-power magnetic sensor node.

For outdoor deployment, the electronic and power-supply components were installed in a compact protective enclosure, as shown in Figure 3. The enclosure separates the sensing electronics from the higher-capacity battery pack and protects the system against mechanical damage and environmental exposure. Cable glands are used for external sensor, communication, charging, or control connections while maintaining the enclosure integrity. The compact housing enables the complete sensor to be mounted directly on existing roadside infrastructure, such as a guardrail, pole, or traffic-sign support.

The roadside installation does not require drilling into the pavement or interrupting traffic. This significantly simplifies deployment compared with road-embedded magnetic sensors and inductive loops. The enclosure can be attached to the guardrail near the monitored lane and removed after completing the traffic survey. However, because the measured magnetic disturbance decreases with increasing distance from the vehicle, the mounting position and sensor orientation must remain consistent throughout the measurement.

### 2.2. Magnetic Sensing Subsystem

The magnetic sensing subsystem is based on two PNI RM3100 three-axis magnetometers (PNI Sensor, Santa Rosa, CA, USA) installed on opposite sides of the main printed circuit board. Each magnetometer measures the X-, Y-, and Z-axis components of the local magnetic field. When a vehicle passes the measurement point, its ferromagnetic structure perturbs the Earth’s magnetic field and produces a characteristic vector-valued time signature. The RM3100 was retained after the authors’ earlier comparative measurements under the intended roadside traffic-monitoring conditions [12,13,14]. This selection should not be interpreted as a claim that the RM3100 is universally superior to all magnetoresistive technologies. AMR, GMR, and TMR sensors have all been successfully applied to vehicle detection and classification [8,9,11,17], and recent TMR devices in particular can provide high sensitivity and low power consumption. Fluxgate, Hall-effect, and GMI devices were not included in the authors’ original traffic-specific comparison; consequently, the present study does not claim a universal performance ranking of the RM3100 against those technologies. For the present roadside geometry, however, the design prioritised signal clarity and noise robustness at increased lateral distance, together with a wide measurement range, digital I^2^C/SPI interfacing, and three-axis vector output. Manufacturer data for the RM3100 specify a sensitivity of approximately 13 nT, noise of approximately 15 nT under the stated configuration, a field range of approximately ±1100 µT, and an operating-temperature range of −40 °C to +85 °C [18]. A separate in situ characterisation of the complete node noise floor and baseline drift under the deployed 250 Hz operating configuration was not performed in the present study. Consequently, the noise value quoted above is a manufacturer specification rather than a measured system-level noise floor.

Two RM3100 modules were incorporated primarily to provide hardware redundancy during unattended roadside measurements. In normal operation, both sensors can observe the same event, allowing their operational state and responses to be compared and permitting continued acquisition if one sensor becomes unavailable. The main board also includes an external sensor connector for experimental extensions. Magnetic-field components are sampled at 250 Hz (4 ms sampling interval), which provides sufficient temporal resolution for the intended vehicle signatures while limiting bus traffic, buffer size, storage volume, and embedded processing load. The selected rate is therefore an application-specific compromise rather than a universal optimum. The orientation and position of the node are kept consistent during each measurement campaign because signal amplitude and shape depend on vehicle trajectory, lateral distance, speed, and sensor-axis orientation.

The three-axis configuration is used because the vehicle disturbance is inherently vector-valued and its projection on any single axis depends on installation orientation and trajectory. Using all three axes also enables the orientation-independent energy channel employed by the detector and CNN. Previous traffic-classification studies have shown that the selected combination of magnetometer axes can affect classification performance and that single-axis operation can remain feasible, although it provides less vector information [8,9]. A controlled single-axis-versus-three-axis ablation was not performed for the present CNN; consequently, this paper does not claim a quantified accuracy gain attributable solely to the three-axis input. Such an ablation is identified as a useful extension for future work.

## 3. Signal Preprocessing and Vehicle Detection

**Figure 4:**
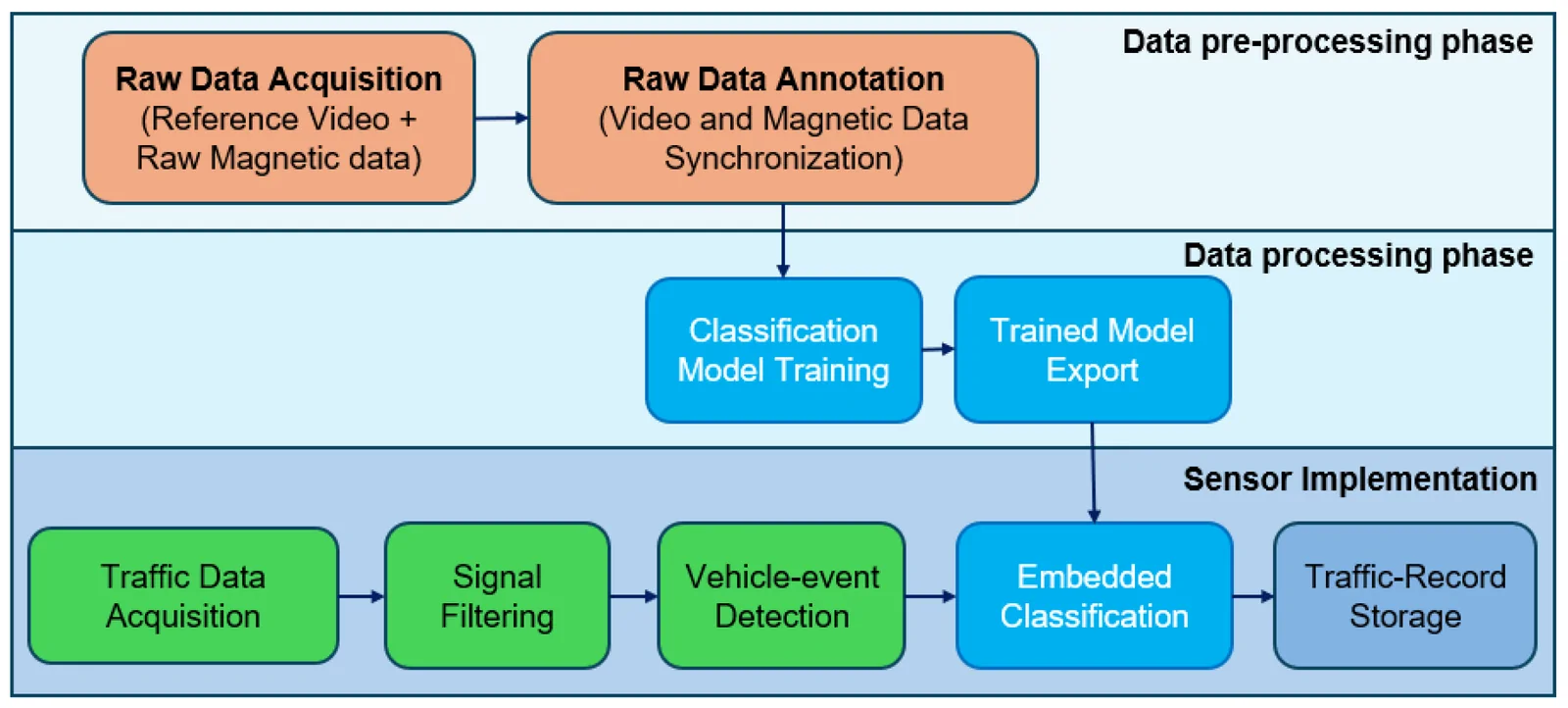
Workflow of magnetic data acquisition, dataset preparation, model training, embedded implementation, and real-time vehicle classification.

The processing workflow of the proposed system consists of two interconnected phases. In the offline development phase, magnetic measurements are synchronised with reference video and radar data, manually verified, and used to train the vehicle-classification models. The selected model is subsequently exported and implemented on the embedded sensing platform. During roadside operation, the sensor node continuously acquires magnetic-field data, performs signal filtering and vehicle-event detection, classifies each detected event, and stores the resulting traffic record. The complete processing workflow is illustrated in Figure 4. The magnetic signal-processing and event-detection procedure follows the methodology previously introduced by the authors in [15]. The study provides a detailed description of data acquisition, multimodal synchronisation, annotation, and dataset generation. In the present work, the same processing principles are transferred to the NXP MK22FN512VLH12 microcontroller and integrated with the embedded vehicle-classification model.

In the time domain, a passing vehicle produces a transient, often multi-lobed deviation of the three magnetic components from their local baselines. Larger vehicles frequently produce stronger or more complex signatures, but signal amplitude is not a direct proxy for vehicle mass because ferromagnetic-material distribution, lateral distance, speed, orientation, and trajectory also influence the measured waveform [8,9,15,17]. The preprocessing chain below therefore removes slow baseline variation, suppresses high-frequency noise, and forms an energy-based detection signal before a fixed-length event window is presented to the classifier. Representative magnetic signatures and the underlying annotation workflow for this dataset were reported previously in [15]. The present paper focuses on their embedded processing and classification rather than repeating the dataset-generation experiments. Representative examples of the actual measured signals are available in the authors’ earlier studies, specifically Refs. [14,15]. These studies provide representative measured examples of real vehicle-induced magnetic disturbances, including the RM3100 response and the effect of sensor-to-lane distance, as well as raw magnetic flux-density data, the filtered/windowed energy signal, and the filtered event waveform. These previously published signal plots are not duplicated here because the present paper focuses on the embedded implementation of the same sensing and preprocessing chain.

The raw magnetic-field signals measured along the three sensor axes are denoted by $x\left[n\right]$, $y\left[n\right]$, and $z\left[n\right]$. Each signal contains a static component of the Earth’s magnetic field, slowly varying baseline changes, vehicle-induced disturbances, and high-frequency measurement noise. The same two-stage filtering procedure is applied independently to each magnetic-field axis.

Let $a\left[n\right]$, where $a\in \left\{x,y,z\right\}$ denote the raw signal of one magnetic-field axis. First, the slowly varying baseline is removed by subtracting a moving average calculated from the preceding $N_{HP}$ samples:

(1) $$a_{HP}\left[n\right]=a\left[n\right]-\frac{1}{N_{HP}}\sum_{k=n-N_{HP}+1}^{n}a\left[k\right],a\in \left\{x,y,z\right\}$$

Here, $a_{HP}\left[n\right]$ denotes the high-pass-filtered signal of the corresponding axis. This operation suppresses the static and slowly varying magnetic-field components while preserving transient disturbances caused by passing vehicles.

The high-pass-filtered signal is subsequently smoothed using an exponential low-pass filter:

(2) $$a_{LP}\left[n\right]=\alpha a_{HP}\left[n\right]+\left(1-\alpha \right)a_{LP}\left[n-1\right],0<\alpha \leq 1$$

where $a_{LP}\left[n\right]$ is the filtered signal and α is the smoothing coefficient. Lower values of α provide stronger smoothing, whereas higher values preserve faster variations in the measured magnetic signature.

The instantaneous energy of the filtered three-axis magnetic signal is calculated as

(3) $$e\left[n\right]=x_{LP}^{2}\left[n\right]+y_{LP}^{2}\left[n\right]+z_{LP}^{2}\left[n\right]$$

To emphasise vehicle-induced disturbances and suppress isolated fluctuations, the signal energy is accumulated over a sliding window containing M samples:

(4) $$E\left[n\right]=\sum_{k=n-M+1}^{n}e\left[k\right]$$

The resulting energy signal is independent of signal polarity and highlights intervals during which the measured magnetic field differs significantly from its background level. Vehicle passages are detected by searching for local maxima in $E\left[n\right]$. Let $n_{p}$ denote the sample index of a candidate local maximum and $n_{p-1}$ the index of the previously accepted event. A candidate peak is accepted when

(5) $$E\left[n_{p}\right]\geq m,n_{p}-n_{p-1}\geq d,N_{above}\geq N_{t}$$

where m is the minimum peak magnitude, d is the minimum distance between two consecutively accepted peaks, $N_{above}$ is the number of consecutive samples for which the energy signal remains above the reference threshold, and $N_{t}$ is the required minimum duration expressed in samples.

If the minimum duration t is specified in milliseconds, the corresponding number of samples is calculated as

(6) $$N_{t}=\left\lceil \frac{tf_{s}}{1000}\right\rceil$$

After a valid event has been detected, a fixed-length window containing $N_{w}=512$ samples is extracted around the corresponding energy maximum. The extracted four-channel event representation is

(7) $$X_{p}=\left[\begin{array}{ccc} x_{LP}\left[n_{s}\right] & \cdots & x_{LP}\left[n_{e}\right]\\ y_{LP}\left[n_{s}\right] & \cdots & y_{LP}\left[n_{e}\right]\\ z_{LP}\left[n_{s}\right] & \cdots & z_{LP}\left[n_{e}\right]\\ e\left[n_{s}\right] & \cdots & e\left[n_{e}\right] \end{array}\right],X_{p}\in R^{4\times 512}$$

where

(8) $$n_{s}=n_{p}-\left\lfloor \frac{N_{w}}{2}\right\rfloor ,n_{e}=n_{s}+N_{w}-1$$

At the sampling frequency $f_{s}=250\;Hz$, the temporal duration of the extracted event window is

(9) $$T_{w}=\frac{N_{w}}{f_{s}}=\frac{512}{250}=2.048\;s$$

The fixed-length representation ensures that all detected events have the same input dimensions. During embedded operation, signal filtering and event detection are executed continuously, whereas the neural network classifier is activated only after a complete valid event window has been acquired.

### 3.1. Dataset Preparation and Vehicle Classes

The dataset used for the development of the vehicle-classification model was created using the measurement and annotation methodology described in detail in the authors’ previous study [15]. The present paper does not introduce the dataset-generation procedure as a new contribution; instead, it uses the previously created and manually verified dataset to develop and evaluate the classification model implemented on the embedded sensing platform.

More than 36 h of real-world traffic measurements were collected at eight locations in the Žilina region. The experimental setup combined the RM3100 magnetometer with a high-definition video camera and, where available, a Sierzega SR4 traffic radar. The magnetic measurements were temporally synchronised with the reference video and radar data, allowing each detected magnetic event to be associated with the corresponding vehicle and its verified category. The complete acquisition, synchronisation, annotation, and verification workflow is described in [15]. The initial processing pipeline generated approximately 150,000 candidate events. These records included correctly detected vehicles, traffic in the opposite lane, noise-induced detections, incomplete signatures, and other ambiguous events. Following signal filtering, synchronisation with the reference data, and manual verification, approximately 50,000 valid and labelled records were retained for subsequent analysis.

Each annotated event was assigned to one of five predefined classes. Class 1 contains motorcycles and passenger cars. Class 2 includes vans, delivery vehicles, and light-duty trucks with a gross vehicle weight of up to 3.5 t. Class 3 comprises standard trucks and buses, whereas Class 4 represents heavy-duty trucks and articulated vehicle combinations. Class 0 contains non-vehicle detections, measurement noise, opposite-lane events, and ambiguous records that could not be reliably assigned to one of the vehicle categories. The numbers of records in the individual classes are summarised in Table 1.

For classifier development, the original dataset labels were transformed into four operational classes and one auxiliary rejection class. Operational Class C0 contains events without a relevant vehicle, including noise-induced and ambiguous detections. Operational Class C1 contains motorcycles and passenger cars. Operational Class C2 combines vans, delivery vehicles, light-duty trucks, standard trucks, and buses. Operational Class C3 contains heavy-duty trucks and articulated vehicle combinations. Opposite-direction events were separated from the original non-target records and assigned to an additional rejection output. This fifth output is used by the embedded classifier to reject vehicles travelling in the opposite direction and is not included in the reported four-class confusion matrices. The mapping of the original dataset annotations to the operational classes and the auxiliary rejection output is summarised in Table 2.

The annotated magnetic signatures were subsequently divided into training and testing subsets for model development. In the preliminary evaluation, 80% of the available records were used for model training and the remaining 20% for testing. The same dataset partition was used to compare the investigated classification approaches under identical conditions.

### 3.2. Classification Model Development

Following vehicle-event detection and extraction of the corresponding magnetic signature, the classification stage determines the operational category of the detected event. The four principal classes C0–C3 are defined in Table 2. In addition, the embedded neural network contains a fifth output dedicated to opposite-direction vehicles. Events assigned to this output are rejected and excluded from the resulting traffic statistics.

Before selecting the model for embedded implementation, several supervised machine learning approaches were evaluated using the MATLAB v21.2 Classification Learner application. The investigated classifiers included Gaussian Naive Bayes, a decision tree, k-nearest neighbours (KNN), and a support vector machine (SVM). These methods were selected to compare probabilistic, rule-based, distance-based, and margin-based classification principles using the same magnetic-signature dataset. The available labelled data were divided into a training subset containing 80% of the samples and an independent testing subset containing the remaining 20%. The training subset was used to fit the individual classification models, while the testing subset was used exclusively for performance evaluation. All investigated classifiers were therefore evaluated under the same data-splitting conditions.

The classification results were evaluated using confusion matrices. Let $n_{ij}$ denote the number of samples belonging to the true class $C_{i}$ that were assigned by the classifier to class $C_{j}$. The diagonal elements $n_{ii}$ therefore represent correctly classified samples, whereas the off-diagonal elements represent misclassifications. The overall classification accuracy was calculated as

(10) $$OA=\frac{\sum_{i=0}^{K-1}n_{ii}}{\sum_{i=0}^{K-1}\sum_{j=0}^{K-1}n_{ij}}\cdot 100\%$$

Here, K=4 denotes the number of considered classes.

Because the dataset was imbalanced, the overall accuracy was supplemented by class-specific recall and precision. Recall represents the proportion of samples belonging to class $C_{i}$ that were correctly classified:

(11) $${Recall}_{i}=\frac{n_{ii}}{\sum_{j=0}^{K-1}n_{ij}}\cdot 100\%$$

Precision, also referred to as the positive predictive value, represents the proportion of samples assigned to class $C_{i}$ that actually belonged to that class:

(12) $${Precision}_{i}=\frac{n_{ii}}{\sum_{j=0}^{K-1}n_{ji}}\cdot 100\%$$

These metrics were used jointly to compare the investigated classification approaches and identify systematic confusion between the individual vehicle categories.

#### 3.2.1. Gaussian Naive Bayes Classifier

The first evaluated approach was the Gaussian Naive Bayes classifier. This probabilistic method applies Bayes’ theorem and assumes that the individual input features are conditionally independent within each class. For an input feature vector $x={\left[x_{1},x_{2},\ldots ,x_{D}\right]}^{T},$ the posterior probability of class $C_{k}$ is expressed as

(13) $$P\left(C_{k}|x\right)=\frac{P\left(C_{k}\right)\;P\left(x|C_{k}\right)}{P\left(x\right)}$$

Under the conditional-independence assumption, the class-conditional probability can be written as

(14) $$P\left(x|C_{k}\right)=\prod_{d=1}^{D}P\left(x_{d}|C_{k}\right)$$

In the Gaussian variant, the probability distribution of each feature $x_{d}$ within class $C_{k}$ is approximated by a normal distribution:

(15) $$P\left(x_{d}|C_{k}\right)=\frac{1}{\sqrt{2\pi \sigma_{kd}^{2}}}\exp\left[-\frac{{\left(x_{d}-\mu_{kd}\right)}^{2}}{2\sigma_{kd}^{2}}\right]$$

where $\mu_{kd}$ and $\sigma_{kd}^{2}$ denote the mean and variance of feature d estimated from the training samples belonging to class $C_{k}$, respectively. The predicted class is then determined as

(16) $$\hat{C}={\arg\;\max}_{C_{k}}\;\left[P\left(C_{k}\right)\prod_{d=1}^{D}P\left(x_{d}|C_{k}\right)\right]$$

**Figure 5:**
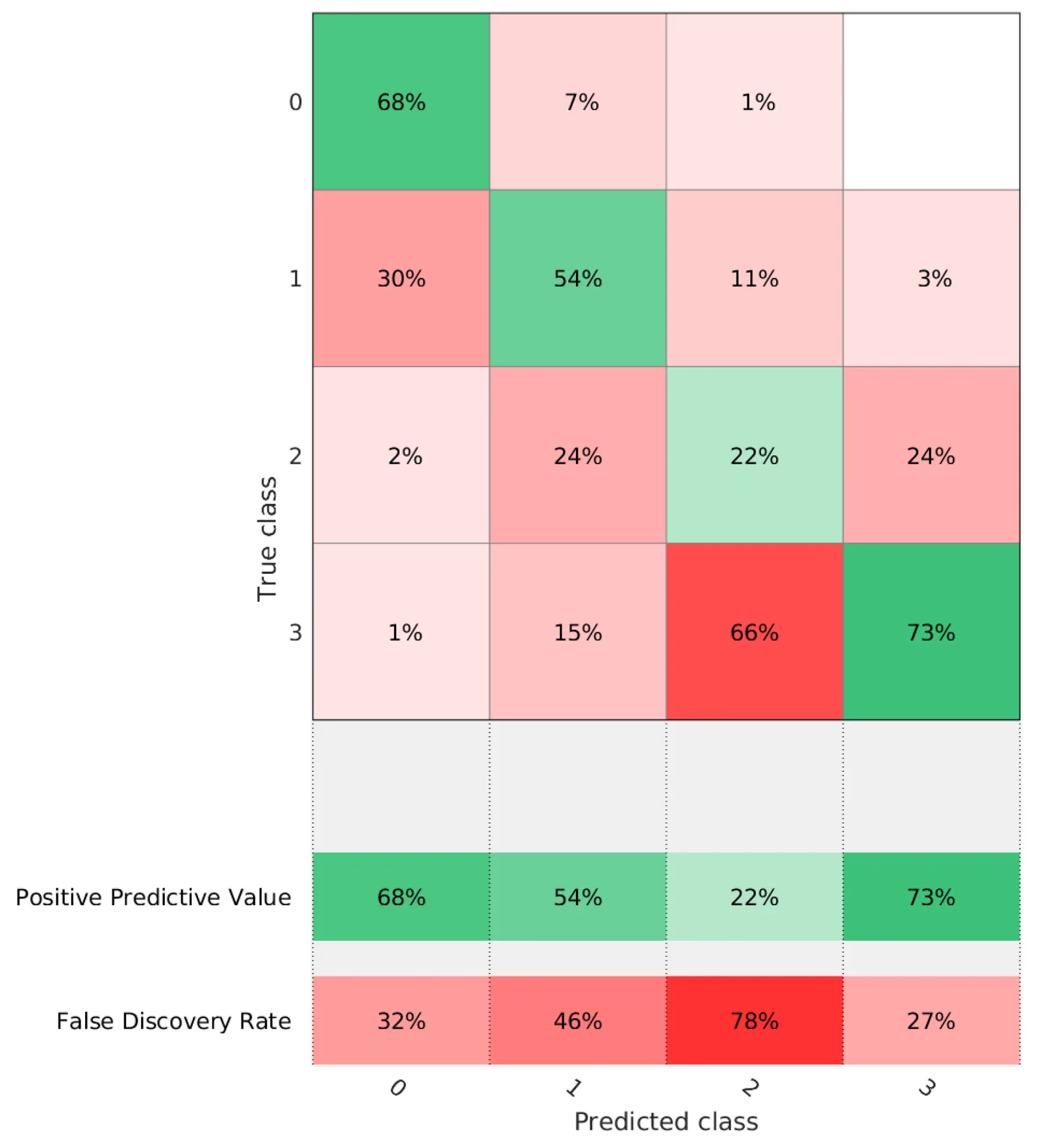
Confusion matrix of the Gaussian Naive Bayes classifier evaluated using the 20% testing subset. The columns represent predicted classes, while the rows represent true classes. Green shading denotes correctly classified/positive-predictive-value cells, whereas red shading denotes misclassification/false-discovery components; darker shades indicate larger percentages.

Equations (13)–(15) define how the Gaussian Naive Bayes model converts an input feature vector into class-specific posterior scores, and Equation (16) selects the class with the largest score. These equations are applied independently to every sample in the 20% testing subset. Figure 5 is therefore not a direct graphical representation of Equations (13)–(16); rather, it is the aggregate evaluation obtained after the class predicted by Equation (16) for each test sample is compared with its ground-truth label. The matrix is normalised by predicted class, so each column represents the composition of one predicted category, and its diagonal value equals the corresponding precision (positive predictive value). Under this normalisation, the classifier achieved precision values of 68% for C0, 54% for C1, 22% for C2, and 73% for C3. This explicit link between the probabilistic decision rule and the confusion matrix clarifies how the equations lead to the results shown in Figure 5.

The results indicate that the Gaussian Naive Bayes classifier was most reliable when identifying Class C3, representing heavy-duty and articulated vehicles. However, the precision of Class C2 was only 22%, with a corresponding false discovery rate of 78%. Most samples assigned to C2 originated from Class C3, which accounted for 66% of all predictions in this column. This result indicates a substantial overlap between the magnetic signatures of medium-sized and heavy vehicles. The classifier also exhibited difficulties in distinguishing Class C1 from the other categories. Only 54% of the samples assigned to C1 actually belonged to this class, whereas 24% originated from C2 and 15% from C3. Furthermore, 30% of the samples classified as C0 were actually C1 events. These errors show that the conditional-independence and Gaussian-distribution assumptions did not adequately represent the relationships present in the magnetic signatures. Although the method has low computational complexity and modest memory requirements, its limited separation of neighbouring vehicle categories makes it unsuitable as the final classifier for the proposed embedded system.

#### 3.2.2. Decision Tree Classifier

The second evaluated approach was a decision tree classifier. This method recursively divides the input feature space into a set of regions using a sequence of binary decision rules. At each internal node, one input feature is compared with a selected threshold, and the sample is subsequently directed to one of two branches:

(17) $$x_{d}\leq \theta \;\mathrm{or}\;x_{d}>\theta ,$$

where $x_{d}$ denotes the evaluated feature and θ is the corresponding decision threshold. This process continues until the sample reaches a terminal leaf containing the predicted class. The impurity of a node can be expressed using the Gini index:

(18) $$G\left(t\right)=1-\sum_{k=0}^{K-1}p_{k,t}^{\;2},$$

where $p_{k,t}$ is the proportion of training samples belonging to class $C_{k}$ at node t, and K=4 is the number of classes. A suitable split reduces the weighted impurity of the resulting child nodes. The class assigned to the terminal leaf t is determined as

(19) $$\hat{C}\left(t\right)={\arg\;\max}_{k}|p_{k,t}.$$

**Figure 6:**
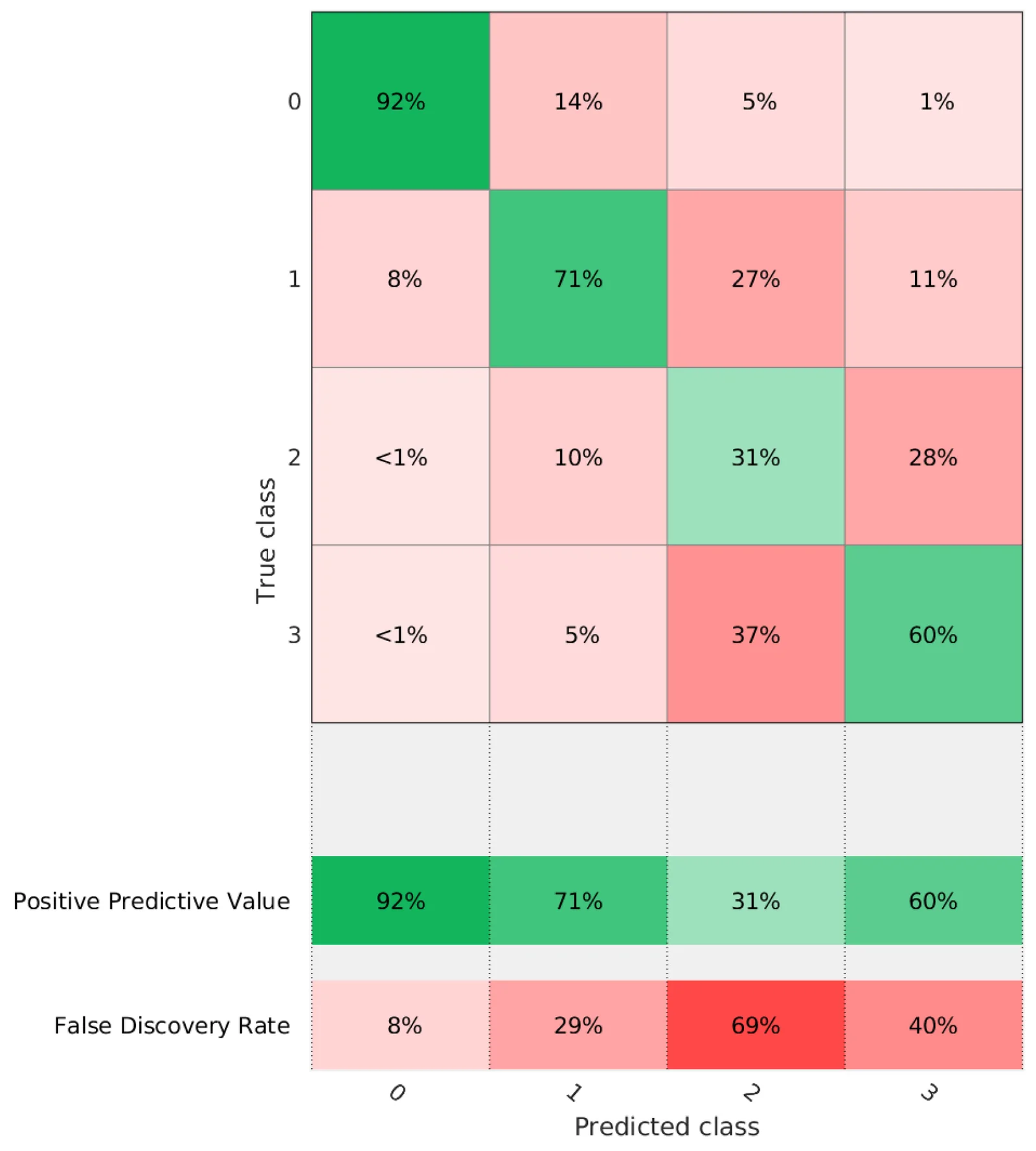
Confusion matrix of the decision tree classifier evaluated using the 20% testing subset. The columns represent predicted classes, while the rows represent true classes.

The confusion matrix obtained for the decision tree classifier is presented in Figure 6. As in the previous evaluation, the matrix is normalised according to the predicted class, and the diagonal elements therefore represent the precision achieved for the individual categories.

The decision tree achieved precision values of 92% for Class C0, 71% for Class C1, 31% for Class C2, and 60% for Class C3. Compared with the Gaussian Naive Bayes classifier, the decision tree provided a considerable improvement for Classes C0, C1, and C2. In particular, the precision of C0 increased from 68% to 92%, indicating that the tree-based decision rules were more effective in separating irrelevant events from valid vehicle passages. The precision of C1 also increased from 54% to 71%. Nevertheless, substantial confusion remained between Classes C2 and C3. Only 31% of the samples assigned to C2 actually belonged to this category. The remaining C2 predictions consisted mainly of C3 samples, accounting for 37%, and C1 samples, accounting for 27%. Similarly, among the samples predicted as C3, 28% belonged to C2 and 11% belonged to C1. The precision of C3 consequently reached only 60%, which was lower than the 73% obtained using Gaussian Naive Bayes. The results demonstrate that the nonlinear and rule-based structure of the decision tree represented the magnetic signatures more effectively than the probabilistic assumptions of Gaussian Naive Bayes. However, the model still provided insufficient discrimination between medium-sized and heavy vehicles. Its classification performance can also be sensitive to the selected tree depth and may deteriorate if the tree becomes overfitted to the training data. Therefore, the decision tree was retained as a useful low-complexity reference model but was not selected for final embedded deployment.

#### 3.2.3. K-Nearest Neighbours Classifier

The third evaluated approach was the k-nearest neighbours (KNN) classifier. Unlike probabilistic and tree-based methods, KNN does not construct an explicit parametric model during training. Instead, it stores the labelled training samples and determines the class of a new input according to the classes of the most similar samples in the training dataset.

For two feature vectors x and z, their distance can generally be expressed using the Minkowski metric:

(20) $$d\left(x,z\right)={\left(\sum_{m=1}^{D}{|x_{m}-z_{m}|}^{p}\right)}^{1/p}$$

where D is the number of input features and p determines the selected distance metric. For p = 2, Equation (20) corresponds to the Euclidean distance.

**Figure 7:**
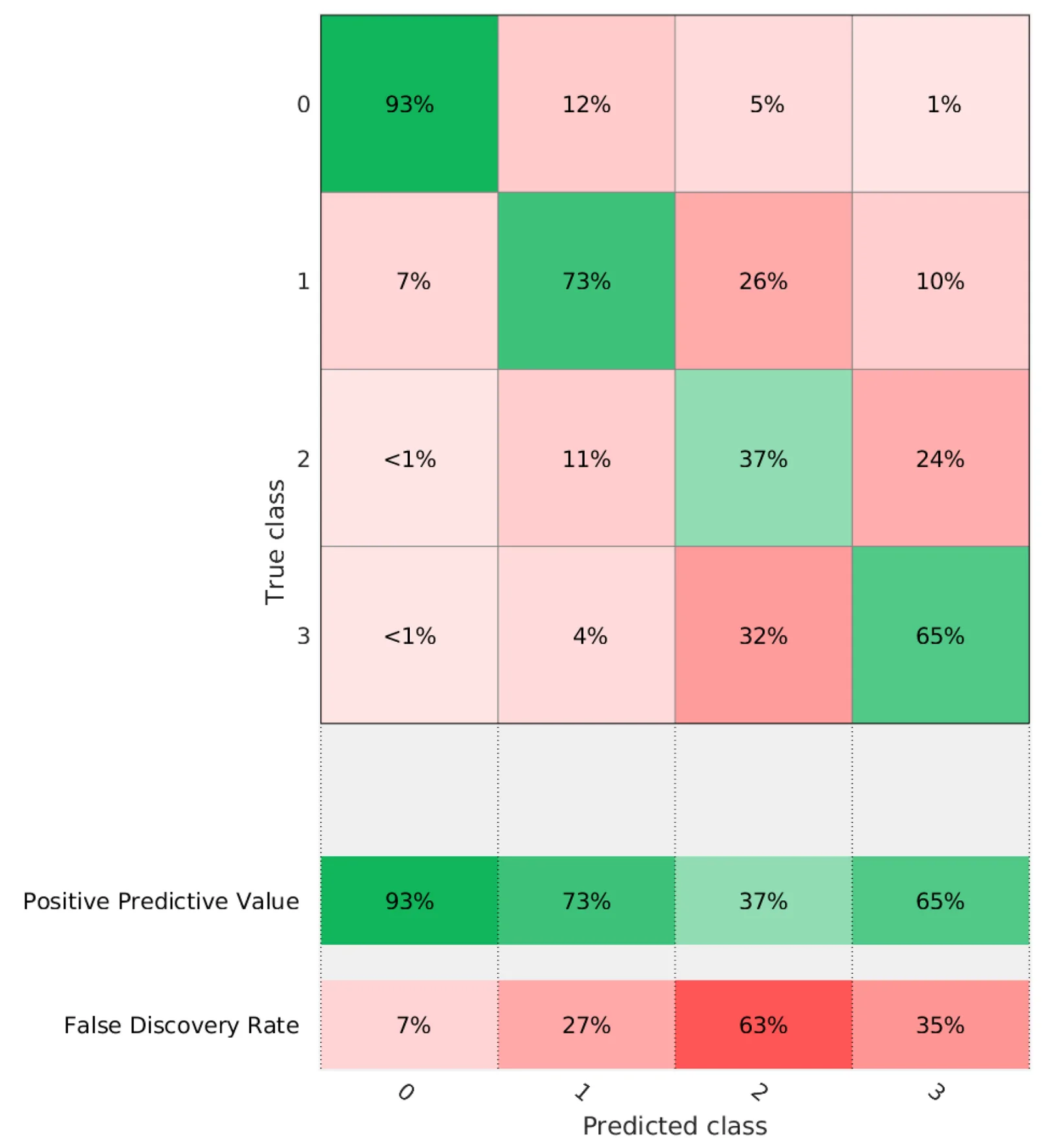
Confusion matrix of the KNN classifier evaluated using the 20% testing subset. The columns represent predicted classes, while the rows represent true classes.

Let $N_{k}\left(x\right)$ denote the set of k training samples closest to the input vector x. In the standard majority-voting formulation, the predicted class is determined as

(21) $$\hat{C}={\arg\;\max}_{C_{c}}\;\sum_{x_{i}\in N_{k}\left(x\right)}I\left(y_{i}=C_{c}\right)$$

where $y_{i}$ is the class label of the i-th neighbouring sample and $I\left(\cdot \right)$ is an indicator function that equals one when the stated condition is satisfied and zero otherwise. The confusion matrix obtained for the KNN classifier is shown in Figure 7.

The KNN classifier achieved precision values of 93% for Class C0, 73% for Class C1, 37% for Class C2, and 65% for Class C3. It therefore provided a moderate improvement over the decision tree for all four categories. The highest precision was obtained for C0, demonstrating reliable separation of irrelevant events and valid vehicle passages. Class C1 also achieved a precision of 73%, although 12% of the samples assigned to this category originated from C0 and 11% originated from C2. Class C2 remained the most difficult category to identify. Only 37% of the samples assigned to C2 actually belonged to this class, while 32% originated from C3 and 26% from C1. This result confirms that the magnetic signatures of neighbouring vehicle categories overlap considerably. For C3 predictions, 65% were correct, whereas 24% belonged to C2 and 10% to C1.

Overall, KNN provided better class separation than Gaussian Naive Bayes and the decision tree, particularly for the intermediate C2 category. Nevertheless, its direct implementation on a resource-constrained microcontroller is problematic because the method requires storage of the reference training samples and repeated distance calculations during every inference. Its memory requirements and inference time also increase with the size of the training dataset. KNN was therefore used as a comparative offline model but was not considered suitable for deployment on the proposed low-power embedded platform.

#### 3.2.4. Support Vector Machine Classifier

The fourth evaluated approach was the support vector machine (SVM). An SVM constructs a decision boundary that maximises the separation margin between samples belonging to different classes. For a binary classification problem, the decision function can be expressed as

(22) $$f\left(x\right)=w^{T}\phi \left(x\right)+b$$

where x is the input feature vector, $\phi \left(x\right)$ denotes its transformation into the selected feature space, w is the normal vector of the separating hyperplane, and b is the bias term. The predicted binary class is determined according to the sign of the decision function:

(23) $$\hat{y}=\mathrm{sign}\left[f\left(x\right)\right]$$

**Figure 8:**
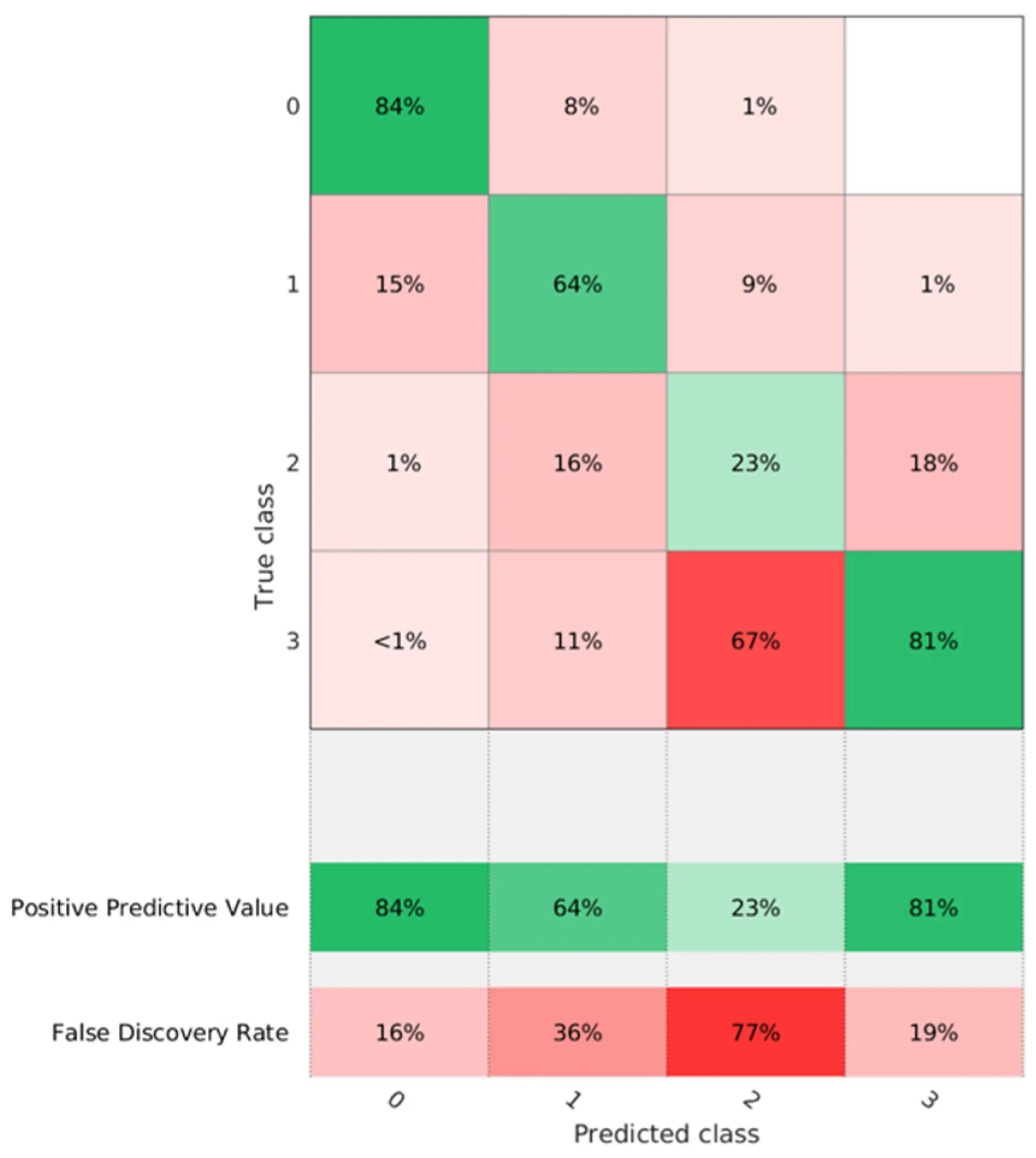
Confusion matrix of the SVM classifier evaluated using the 20% testing subset. The columns represent predicted classes, while the rows represent true classes.

The parameters of the separating hyperplane are obtained by solving the soft-margin optimisation problem

(24) $$\underset{w,b,\xi }{min}\left(\frac{1}{2}∥w{∥}^{2}+C\sum_{i=1}^{N}\xi_{i}\right)$$

subject to

(25) $$y_{i}\left[w^{T}\phi \left(x_{i}\right)+b\right]\geq 1-\xi_{i},\xi_{i}\geq 0,$$

where C is the regularisation parameter, $\xi_{i}$ is the slack variable associated with the i-th training sample, and N is the number of training samples. For the considered four-class problem, multiple binary decision functions were combined to obtain the final multiclass prediction. The confusion matrix obtained for the SVM classifier is presented in Figure 8.

The SVM classifier achieved precision values of 84% for Class C0, 64% for Class C1, 23% for Class C2, and 81% for Class C3. Its strongest result was obtained for C3, for which it provided the highest precision among the four conventional classification approaches. Only 18% of the samples assigned to C3 originated from C2, while approximately 1% originated from C1. In contrast, the SVM performed poorly for Class C2. Only 23% of the samples predicted as C2 actually belonged to this category, resulting in a false discovery rate of 77%. As many as 67% of the samples assigned to C2 belonged to C3. This indicates that the SVM decision boundaries did not provide sufficient separation between the magnetic signatures of medium-sized and heavy vehicles.

The precision of C0 and C1 reached 84% and 64%, respectively, which was lower than the corresponding values obtained using the decision tree and KNN classifiers. In particular, 15% of the samples classified as C0 actually belonged to C1. Similarly, predictions assigned to C1 included samples from all remaining categories. Although the SVM provided good precision for heavy vehicles, its performance was not balanced across the four classes. Moreover, embedded deployment of a multiclass SVM may require the storage of multiple decision functions and support vectors, with computational and memory requirements depending on the selected kernel and the number of support vectors. The SVM was therefore not selected as the final model for implementation on the MK22 microcontroller.

#### 3.2.5. One-Dimensional Convolutional Neural Network Classifier

Based on the limitations observed for the conventional classifiers, a compact one-dimensional convolutional neural network (1D CNN) was developed for embedded implementation. The model was designed to process the temporal structure of the magnetic signatures while maintaining computational and memory requirements compatible with the MK22 microcontroller.

The neural network input consists of four synchronised channels, each containing 512 samples. The first three channels represent the preprocessed X-, Y-, and Z-axis magnetic-field signals. The fourth channel represents the combined instantaneous signal energy calculated as

(26) $$e\left[n\right]=x^{2}\left[n\right]+y^{2}\left[n\right]+z^{2}\left[n\right],n=1,\ldots ,512.$$

The complete input tensor is therefore expressed as

(27) $$X=\left[\begin{array}{cccc} x\left[1\right] & x\left[2\right] & \cdots & x\left[512\right]\\ y\left[1\right] & y\left[2\right] & \cdots & y\left[512\right]\\ z\left[1\right] & z\left[2\right] & \cdots & z\left[512\right]\\ e\left[1\right] & e\left[2\right] & \cdots & e\left[512\right] \end{array}\right]\in R^{4\times 512}$$

The network comprises three consecutive one-dimensional convolutional layers followed by a fully connected output layer. For convolutional layer l, the output of filter k at temporal position m is calculated as

(28) $$h_{k}^{\left(l\right)}\left[m\right]=\varphi \left(b_{k}^{\left(l\right)}+\sum_{c=1}^{C_{l-1}}\sum_{r=0}^{K_{l}-1}w_{k,c,r}^{\left(l\right)}h_{c}^{\left(l-1\right)}\left[mS_{l}+r\right]\right)$$

where $C_{l-1}$ is the number of input channels, $K_{l}$ is the convolutional-kernel length, $S_{l}$ is the stride, $w_{k,c,r}^{\left(l\right)}$ and $b_{k}^{\left(l\right)}$ are the corresponding weight and bias parameters, and $\varphi \left(\cdot \right)$ denotes the rectified linear unit activation function.

All three convolutional layers use a kernel length of $K_{l}=8$, a stride of $S_{l}=4$, and no zero padding. The output length of each layer is therefore determined as

(29) $$L_{l}=\left\lfloor \frac{L_{l-1}-K_{l}}{S_{l}}\right\rfloor +1$$

The first convolutional layer transforms the four-channel input into 16 feature maps with a length of 127:

4×512→16×127

The second convolutional layer generates 32 feature maps with a length of 30:

16×127→32×30

The third convolutional layer produces 64 feature maps with a length of 6:

32×30→64×6

The output of the final convolutional layer is flattened into a feature vector containing 64·6=384 elements. This vector is processed by a fully connected layer producing five output scores:

(30) $$y=W_{out}h+b_{out},y\in R^{5}.$$

The first four outputs correspond to the operational classes C0–C3: C0 represents events without a relevant vehicle, C1 represents motorcycles and passenger cars, C2 represents vans and trucks, and C3 represents heavy trucks and articulated vehicle combinations. The fifth output represents a vehicle travelling in the opposite direction and is treated as a rejection output. Events assigned to this output are ignored and are not included in the traffic-category statistics.

The predicted output is determined from the maximum network score:

(31) $$\hat{k}={\arg\;\max}_{k}\;\left(y_{k}\right)$$

If $\hat{k}=4$, the detected event is identified as an opposite-direction vehicle and is rejected. Otherwise, the event is assigned to the corresponding operational category C0–C3. The implemented network requires 288,128 multiply–accumulate operations for one inference. Its fixed computational structure and integer-compatible architecture make it suitable for deterministic execution on the resource-constrained MK22 microcontroller.

**Figure 9:**
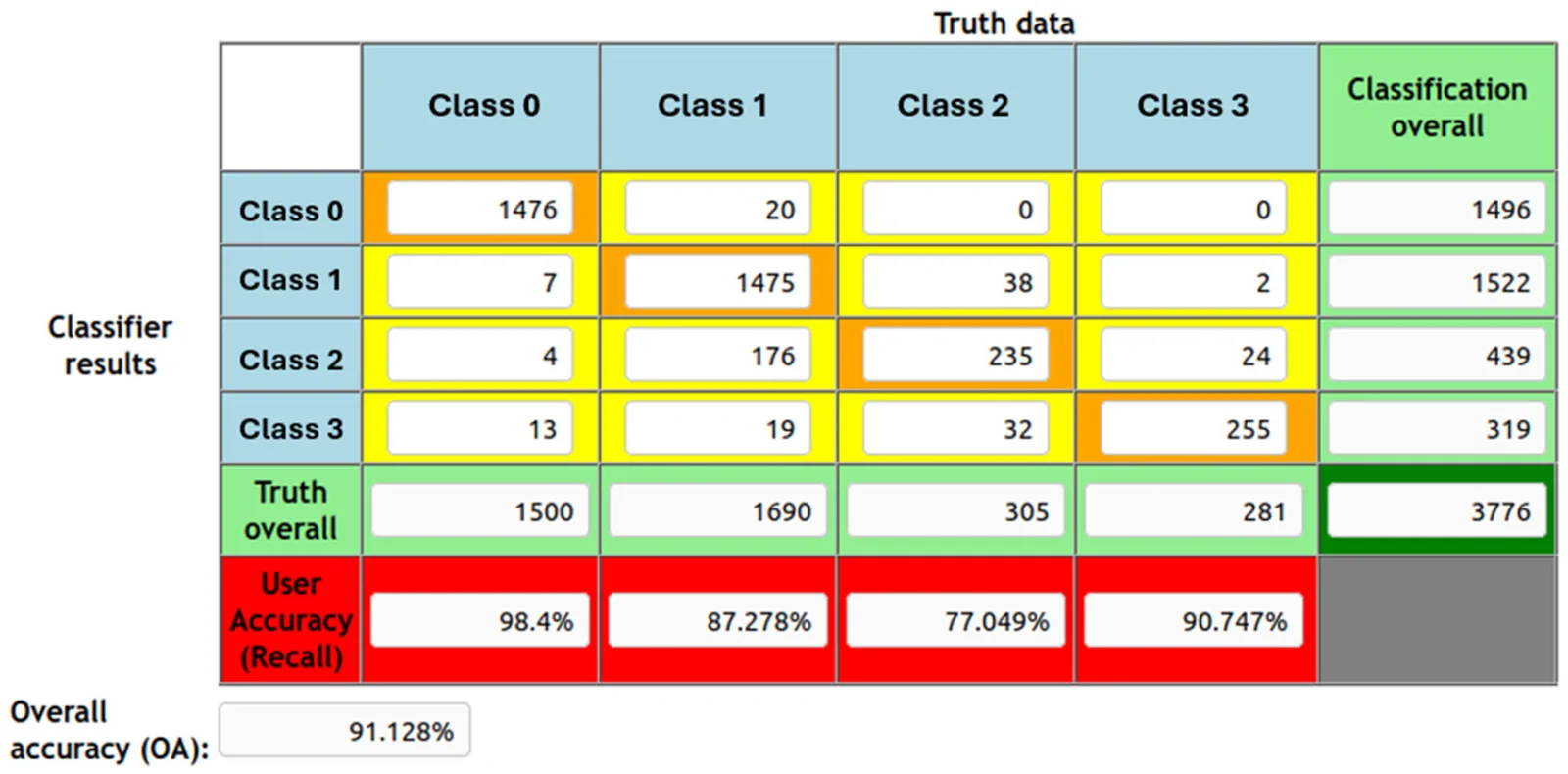
Confusion matrix of the one-dimensional convolutional neural network.

The neural network was evaluated using a testing subset containing 3776 samples from the four operational classes C0–C3. Opposite-direction events assigned to the fifth output were excluded from the confusion matrix presented in Figure 9.

For Class C0, the network correctly classified 1476 of the 1500 testing samples, corresponding to a recall of

(32) $${Recall}_{C0}=\frac{1476}{1500}\cdot 100\%=98.4\%.$$

For Class C1, 1475 of the 1690 samples were classified correctly:

(33) $${Recall}_{C1}=\frac{1475}{1690}\cdot 100\%=87.3\%.$$

For Class C2, the network correctly classified 235 of the 305 testing samples:

(34) $${Recall}_{C2}=\frac{235}{305}\cdot 100\%=77.0\%.$$

Finally, 255 of the 281 Class C3 samples were correctly identified:

(35) $${Recall}_{C3}=\frac{255}{281}\cdot 100\%=90.7\%.$$

The overall accuracy was calculated from the sum of the correctly classified samples:

(36) $$OA=\frac{1476+1475+235+255}{3776}\cdot 100\%=91.1\%.$$

The neural network achieved the highest recall for C0, correctly identifying 1476 of the 1500 testing events without a relevant vehicle. This corresponds to a class-specific recall of 98.4% and demonstrates reliable rejection of non-vehicle and ambiguous detections. The lowest recall was obtained for C2, confirming that vans and trucks represent the most difficult operational category because their magnetic signatures overlap with those of passenger cars and heavy vehicles. Of the 70 incorrectly classified C2 samples, 38 were assigned to C1 and 32 to C3. The network also provided reliable classification of heavy vehicles, achieving a recall of 90.7% for C3. Most of the incorrectly classified C3 samples were assigned to C2, which is consistent with the gradual transition in vehicle dimensions and ferromagnetic mass between the two categories. Compared with the conventional classification approaches, the neural network provided more balanced performance across the individual classes and achieved an overall accuracy of 91.1%. Its compact, fixed architecture also provides deterministic inference without requiring storage of the training dataset. The trained model was therefore selected for implementation on the MK22 microcontroller. To reduce its memory footprint and computational requirements, the model parameters were converted to an 8-bit integer representation, as described in the following section.

## 4. Embedded Edge-AI Implementation

The complete signal-processing and vehicle-classification pipeline was implemented directly on the NXP MK22FN512VLH12 microcontroller using custom-developed C code. The microcontroller operates at a clock frequency of 120 MHz and continuously acquires three-axis magnetic-field measurements from the RM3100 magnetometer at a sampling frequency of 250 Hz. The corresponding sampling period is

(37) $$T_{s}=\frac{1}{f_{s}}=\frac{1}{250}=4\;ms$$

Vehicle-event detection is performed continuously on the incoming magnetic data. After a valid event has been detected, a fixed-length window containing 512 consecutive samples from each magnetic-field axis is extracted. The fourth input channel is subsequently calculated from the instantaneous energy of the three-axis magnetic signal according to Equation (26). The complete input to the neural network is therefore represented by

(38) $$X=\left[\begin{array}{cccc} x\left[1\right] & x\left[2\right] & \cdots & x\left[512\right]\\ y\left[1\right] & y\left[2\right] & \cdots & y\left[512\right]\\ z\left[1\right] & z\left[2\right] & \cdots & z\left[512\right]\\ e\left[1\right] & e\left[2\right] & \cdots & e\left[512\right] \end{array}\right]\in R^{4\times 512}$$

At the applied sampling frequency, each input window represents a measurement interval of

(39) $$T_{w}=\frac{512}{250}=2.048\;s$$

Before neural network inference, the four input channels are independently converted to the signed 8-bit numerical range. For a channel $a\in \left\{x,y,z,e\right\}$, the minimum and maximum values within the 512-sample event window are determined as

(40) $$a_{min}=\underset{1\leq n\leq 512}{min}a\left[n\right],a_{max}=\underset{1\leq n\leq 512}{max}a\left[n\right]$$

Each sample is subsequently quantised according to

(41) $$q_{a}\left[n\right]=\mathrm{round}\left[\frac{a\left[n\right]-a_{min}}{a_{max}-a_{min}}\cdot 255-128\right],a\in \left\{x,y,z,e\right\}$$

The resulting value is saturated to the valid signed 8-bit interval:

(42) $$q_{a}\left[n\right]=\mathrm{clip}\left(q_{a}\left[n\right],-128,127\right)$$

The complete quantised input tensor is therefore expressed as

(43) $$Q=\left[\begin{array}{cccc} q_{x}\left[1\right] & q_{x}\left[2\right] & \cdots & q_{x}\left[512\right]\\ q_{y}\left[1\right] & q_{y}\left[2\right] & \cdots & q_{y}\left[512\right]\\ q_{z}\left[1\right] & q_{z}\left[2\right] & \cdots & q_{z}\left[512\right]\\ q_{e}\left[1\right] & q_{e}\left[2\right] & \cdots & q_{e}\left[512\right] \end{array}\right]\in Z^{4\times 512}$$

Independent channel normalisation compensates for differences in signal range and offset while preserving the temporal shape of the individual input channels. The same preprocessing and quantisation procedure was applied during model development and embedded inference.

The custom C inference function processes the quantised input tensor directly on the microcontroller. The implementation stores the network weights and biases as signed 8-bit integers, while convolutional and fully connected operations use 32-bit integer accumulators. The implementation applies rectified linear unit activation functions after each convolutional layer.

The implemented network contains 22,912 weight parameters:

(44) $$16.+\left(64\cdot 32\cdot 8\right)+\left(5\cdot 384\right)=22\;912$$

The network additionally contains 117 bias parameters:

(45) $$N_{b}=16+32+64+5=117$$

Because both the weights and biases are stored as int8 values, the model parameters occupy 23,029 bytes of Flash memory, excluding the inference code, scaling constants, and runtime buffers. One complete inference requires 288,128 multiply–accumulate operations (MACs): 65,024 in the first convolutional layer, 122,880 in the second, 98,304 in the third, and 1920 in the fully connected layer. This count refers to convolutional and fully connected MACs and excludes non-MACs such as activation, saturation, indexing, and data movement. Intermediate activations and processing buffers are allocated in the available SRAM of the MK22. Neural network inference is initiated only after a valid event has been detected and the complete input window has been acquired. Consequently, the computationally more demanding CNN is not executed continuously at the 250 Hz sampling frequency. This event-driven strategy reduces the average computational load during periods without traffic. The exact inference latency was not measured separately and is therefore not reported. The network generates five output scores. The first four correspond to operational classes C0–C3, whereas the fifth represents a vehicle travelling in the opposite direction. Events assigned to the fifth output are rejected and excluded from the resulting traffic statistics. After classification, the complete three-axis magnetic signature, event timestamp, and predicted vehicle category are stored on the SD card. The derived energy channel does not need to be stored because it can be reconstructed from the three recorded magnetic-field axes. For remote monitoring, the XBee interface transmits a compact event message containing the timestamp and predicted vehicle category. Opposite-direction events are ignored and are not transmitted as valid traffic records. Bluetooth Low Energy provides local configuration and access to the stored measurement results. Transmitting only the event timestamp and predicted category substantially reduces wireless communication requirements compared with transmitting complete magnetic-signal windows. At the same time, local storage of the raw three-axis signatures enables subsequent offline verification, error analysis, and further development of the classification model.

## 5. Comparative Evaluation and Discussion

The practical value of the developed prototype was assessed at two levels. First, the prototype was compared with two commercially available devices used for temporary road-traffic surveys—the Sierzega SR4 radar counter and the Vaisala Nu-Metrics NC200 magnetic traffic analyzer—to provide a concrete product-level reference; the main properties are summarised in Table 3. Second, a broader qualitative comparison with camera, LiDAR, radar, acoustic/DAS, and pavement-embedded sensing is provided in Table 4 to position the proposed approach with respect to installation effort, environmental sensitivity, privacy, and processing burden.

The broader comparison indicates why magnetic sensing is attractive for the targeted use case instead because it dominates every sensing technology. Vision can provide the richest semantic description of a traffic scene, while radar provides direct range and velocity and LiDAR provides detailed geometry. These benefits are accompanied by higher data rates, processing requirements, or equipment cost. Acoustic and distributed-acoustic sensing can cover extended sections but are more dependent on the acoustic/fibre installation context. Magnetic sensing produces compact, privacy-preserving signals and can operate with low-power electronics without illumination, but its signal-to-noise ratio and classification robustness are more sensitive to sensor placement and vehicle distance. The proposed system is therefore aimed primarily at temporary or distributed roadside surveys for which low deployment effort and local edge processing are prioritised [1,2,3,4,5,6,7,8,9,10,11]. From a lifecycle perspective, roadside installation avoids pavement cutting and the associated installation/removal operations, whereas long-term costs remain dependent on battery servicing, enclosure durability, calibration, and field maintenance. A formal lifecycle-cost analysis was not performed in the present study.

The Sierzega SR4 provides the most comprehensive set of directly measured traffic parameters. Its roadside radar detects vehicles in both directions and records their speed, length, direction of travel, timestamp, and temporal gap. The recorded vehicle length is subsequently used for classification into four categories. Its principal advantage is non-invasive and rapid roadside installation. However, radar processing requires a direct view of the monitored traffic lane, and the resulting classification is primarily based on estimated vehicle length rather than on a detailed vehicle-specific signal. The manufacturer specifies a speed range of 2–255 km/h, storage for more than 860,000 vehicles, and continuous battery-powered operation exceeding two weeks [19].

The NC200 also provides vehicle count, speed, length, and classification data. In contrast to the SR4, it uses a GMR magnetic sensor based on Vehicle Magnetic Imaging technology and is installed directly in the monitored traffic lane. Its position beneath passing vehicles provides strong magnetic signatures and enables speed and length estimation. However, installation requires personnel to enter the roadway and attach the protective cover to the pavement using mechanical fasteners or suitable adhesive material. This increases installation time, presents a safety risk, and may require temporary traffic restrictions. The NC200 can store up to 300,000 vehicle records and provides up to 21 days of operation using its rechargeable lithium-ion battery [20].

The proposed prototype combines the non-invasive installation of the radar system with the low-data-rate and privacy-preserving characteristics of magnetic sensing. The enclosure can be mounted on a guardrail, delineator post, or roadside pole without modifying the road surface or interrupting traffic. Vehicle classification is based on the locally measured three-axis magnetic signature rather than primarily on estimated vehicle length. For each detected event, the prototype stores the complete three-axis signature, timestamp, and predicted category on the SD card, whereas the XBee interface transmits only the timestamp and classification result. This selective communication reduces wireless traffic while retaining raw signatures for offline verification, error analysis, and model retraining. The estimated component cost is approximately EUR 500; this is a research-prototype hardware estimate and does not include development, calibration, certification, manufacturing, technical support, or commercial software.

The approximately 41-day autonomy reported for the evaluated battery configuration is an engineering estimate rather than the result of a completed uninterrupted full-discharge field endurance test. It is based on the applied battery capacity and the estimated average consumption of the implemented hardware under event-driven operation. Accordingly, the revised manuscript consistently uses the terms “estimated” or “expected” operating period. Actual endurance can differ because of traffic intensity, radio activity and retransmissions, SD-card write frequency, temperature, battery ageing, and the duty cycles of optional interfaces. A full-duration field discharge test is required before the estimate can be treated as a measured lifetime.

System durability must likewise be distinguished from component-level environmental specifications. The enclosure provides mechanical protection and separation of the electronics from the battery pack, and the RM3100 itself is specified by the manufacturer for −40 °C to +85 °C operation [18]. However, the complete prototype has not yet undergone formal IP-rating assessment or long-duration ingress, salt-fog, thermal-cycling, or vibration testing. The present results therefore demonstrate functional roadside deployment, not a certified environmental durability rating for the complete system.

Several limitations define the current operating envelope. The measurements used for development were obtained with typical roadside lateral distances of approximately 0.5–2 m and installation heights of approximately 0.5–1.5 m. A maximum reliable lateral detection range was not systematically characterised; therefore, 2 m should be interpreted as part of the evaluated/typical geometry rather than as a validated maximum. The fixed CNN input window spans 512 samples, i.e., 2.048 s at 250 Hz, but the system does not directly measure physical vehicle length. Vehicle mass is also not measured: the magnetic response is governed by the amount and spatial distribution of ferromagnetic material as well as by distance and trajectory. Vehicle speed changes the temporal scaling of a magnetic signature—faster passages compress the waveform in time—and related magnetic-anomaly studies report an inverse relationship between signature width and speed [17]. The present dataset contains natural speed variability from real traffic, but classification accuracy was not stratified by radar speed in this study. Consequently, no speed-specific accuracy claim is made. The current prototype also monitors one traffic direction and does not directly measure vehicle speed or length. Future validation should therefore quantify classification accuracy as a joint function of speed, lateral distance, installation height, and road geometry.

## 6. Conclusions

This study presented the design and evaluation of a low-power magnetic sensing platform with embedded Edge-AI processing for road traffic monitoring. The principal new contribution is the integration of previously developed magnetic sensing and dataset methods into a custom microcontroller-resident processing chain that performs acquisition, preprocessing, event detection, quantisation, classification, local verification storage, and selective wireless reporting at the roadside. The sensing methodology and dataset-generation procedure reported in [12,13,14,15] enabled prior work and are not claimed as novel contributions of the present manuscript.

The complete processing chain, including magnetic-data acquisition at 250 Hz, signal filtering, vehicle-event detection, extraction of three-axis 512-sample windows, input quantisation, and vehicle classification, was implemented directly on the microcontroller. Several conventional machine learning methods were initially evaluated, including Gaussian Naive Bayes, a decision tree, KNN, and SVM. Their results demonstrated persistent difficulties in separating neighbouring vehicle categories, particularly medium-sized and heavy vehicles. A compact neural network was therefore selected for embedded implementation. The resulting model achieved an overall classification accuracy of 91.1%. The class-specific recall reached 98.4% for events without a relevant vehicle, 87.3% for motorcycles and passenger cars, 77.0% for vans and trucks, and 90.7% for heavy-duty and articulated vehicles. Opposite-direction vehicles were handled using a separate rejection output and were not included in the reported four-class confusion matrix.

The trained neural network was implemented using custom C code and an 8-bit integer representation. Classification is initiated only after a valid magnetic event has been detected, reducing the average computational load compared with continuous model execution. For each event, the device stores the complete three-axis magnetic signature, timestamp, and predicted vehicle category on the SD card, while only the timestamp and classification result are transmitted through the XBee interface. This event-oriented communication strategy reduces the volume of wirelessly transmitted data and supports autonomous operation.

Compared with the evaluated commercial traffic counters, the prototype combines non-invasive roadside installation, expandable local storage, local intelligent processing, and comparatively low research-prototype component cost. Based on the applied battery capacity and estimated average hardware consumption, the expected autonomous operating period is approximately 41 days. This value is an engineering estimate and has not yet been verified by a complete uninterrupted full-discharge field endurance test; it can vary with traffic intensity, communication activity, SD-card use, temperature, and battery condition.

The current prototype nevertheless has several limitations. It monitors a single traffic direction and does not directly measure vehicle speed, physical length, or mass. A maximum reliable lateral detection range was not established beyond the typical 0.5–2 m deployment geometry. Classification performance depends on sensor position, lateral distance, vehicle trajectory, road geometry, and the similarity between deployment conditions and the training data. Accuracy was not stratified by vehicle speed, and a controlled single-axis-versus-three-axis ablation was not performed. In addition, the complete enclosure/electronics assembly has not undergone formal environmental durability qualification, and the intermediate vehicle category remains the most difficult class because of overlapping magnetic signatures.

Future work will focus on extending the dataset across additional road geometries, vehicle populations, speed ranges, lateral distances, and environmental conditions. Planned analyses include classification accuracy versus speed and placement, controlled one-axis/two-axis/three-axis input ablation, fusion of the two redundant magnetometers, adaptive normalisation, automated post-installation calibration, and formal power/endurance and environmental-durability testing of the complete node. Model optimisation, remote model updates, and additional low-power communication technologies will also be investigated to improve robustness and support larger deployments of autonomous magnetic traffic-monitoring nodes.

## Figures and Tables

**Table 1:** Distribution of the annotated magnetic events among the predefined classes based on the dataset introduced in [15].

Class	Vehicle Category	Number of Records
Class 0	Non-vehicle, noise, opposite-lane, or ambiguous events	24,371
Class 1	Motorcycles and passenger cars	18,396
Class 2	Vans, delivery vehicles, and light-duty trucks up to 3.5 t	2214
Class 3	Standard trucks and buses	1467
Class 4	Heavy-duty trucks and articulated vehicle combinations	3112

**Table 2:** Mapping of the original dataset annotations to the four operational classes and the auxiliary opposite-direction rejection output.

Operational Class	Original Dataset Class	Vehicle Category
C0	Class 0	Non-vehicle, noise, or ambiguous events
C1	Class 1	Motorcycles and passenger cars
C2	Classes 2 and 3	Vans, delivery vehicles, and light-duty trucks up to 3.5 t, standard trucks and buses
C3	Class 4	Heavy-duty trucks and articulated vehicle combinations
Rejection Output	Opposite-direction subset of Class 0	Vehicle travelling in the opposite direction

**Table 3:** Comparison of the proposed magnetic traffic-monitoring prototype with the Sierzega SR4 radar counter and the Vaisala Nu-Metrics NC200 magnetic traffic analyzer [19,20].

Parameter	Sierzega SR4	Vaisala Nu-Metrics NC200	Proposed Prototype
Sensing principle	Side-fire Doppler radar	In-roadway GMR magnetic sensor	Roadside three-axis RM3100 magnetometer with Edge AI
Installation	Roadside pole or delineator post	Directly in the traffic lane	Guardrail, roadside pole, or delineator post
Typical distance from lane	Approximately 0.5–2 m	Centre of the monitored lane	Approximately 0.5–2 m
Installation height	Approximately 1 m	Road-surface level	Approximately 0.5–1.5 m
Road-surface intervention	Not required	Required for mechanical attachment	Not required
Monitored directions	One or two directions	One traffic lane	One direction
Recorded information	Timestamp, speed, length, direction, vehicle gap, category	Timestamp, speed, length and category	Timestamp, predicted category and three-axis magnetic signature
Classification principle	Vehicle length	Vehicle length based on magnetic imaging	Edge-AI analysis of magnetic signature
Number of categories	Four	Configurable length categories	Three vehicle categories, one non-vehicle class, and one opposite-direction rejection output
Operating speed range	2–255 km/h	Approximately 13–193 km/h	Speed measurement not implemented
Storage	More than 860,000 vehicle records	Up to 300,000 vehicle records	Removable SD card; capacity depends on the installed card
Nominal battery operation	More than two weeks	Up to 21 days	Approximately 41 days (engineering estimate)
Approximate cost	EUR 1600, including software	EUR 1300, including software	Approximately EUR 500 in prototype components

**Table 4:** Qualitative positioning of major traffic-monitoring sensing technologies with respect to the requirements of temporary roadside surveys [1,2,3,4,5,6,7,8,9,10,11].

Technology	Road Intervention	Environmental Sensitivity	Privacy/Data Burden	Main Trade-Off for Traffic Surveys
Video/camera	None for roadside mounting	Affected by illumination, shadows, visibility, precipitation, and occlusion	Image data; comparatively high processing/storage; privacy considerations	Rich semantic classification and multi-lane coverage, but higher data/computation and scene dependence
LiDAR	None for roadside mounting	Independent of ambient light; performance can degrade with precipitation/occlusion	Dense geometric point clouds; moderate-to-high processing	Detailed geometry and tracking, but comparatively high sensor and processing cost
Radar	None for roadside mounting	Generally robust to illumination and many weather conditions	Low visual-privacy concern; moderate data volume	Direct range/speed measurement; classification commonly relies on kinematics/length or learned radar features
Acoustic/DAS	No pavement cut when suitable acoustic/fibre infrastructure exists	Sensitive to background vibration/noise, coupling, and installation context	No visual identity; data burden depends strongly on architecture	Potentially long spatial coverage, but infrastructure and signal-processing requirements are site dependent
In-road magnetic/inductive	Pavement intervention required	Largely independent of illumination; robust once correctly installed	Low data volume and low visual-privacy concern	Strong local signal and mature counting, but installation/maintenance may require road works
Proposed roadside magnetic	No pavement intervention	Independent of illumination; sensitive to sensor distance, geometry, and nearby ferromagnetic structures	Low-bandwidth signals; edge inference; only compact event results transmitted	Rapid low-power deployment and local classification, but no direct speed/length measurement and placement-dependent accuracy

## Data Availability

The measurement, synchronization, and annotation methodology for the dataset analysed in this study is described in [15]. Representative raw and processed magnetic signals are reported in [14,15]. Further inquiries regarding access to the underlying data used in this study can be directed to the corresponding author.
